# Supplementary material for: Euclasta condylotricha flowers essential oils: A new source of juvenile hormones and its larvicidal activity against Anopheles gambiae s.s. (Diptera: Culicidae)
Source: PLoS One. 2023 Jan 23;18(1):e0278834. doi: 10.1371/journal.pone.0278834 (PMC9870135; doi:10.1371/journal.pone.0278834)
Supplement: S2 File — (DOCX) [file pone.0278834.s002.docx]

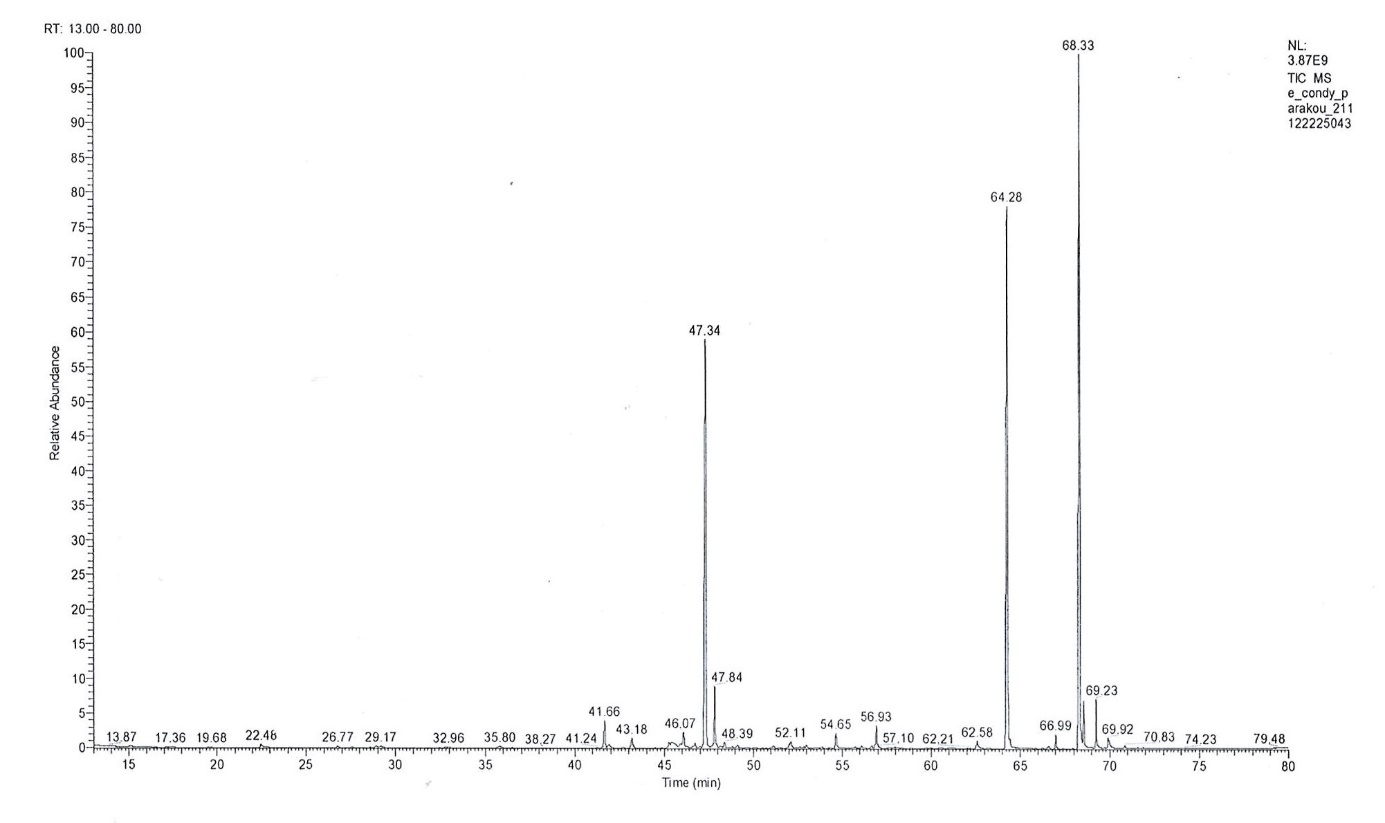


**S1 Fig**: Representative GC–MS chromatogram of Eu. Condylotricha flowers essential oil from Parakou


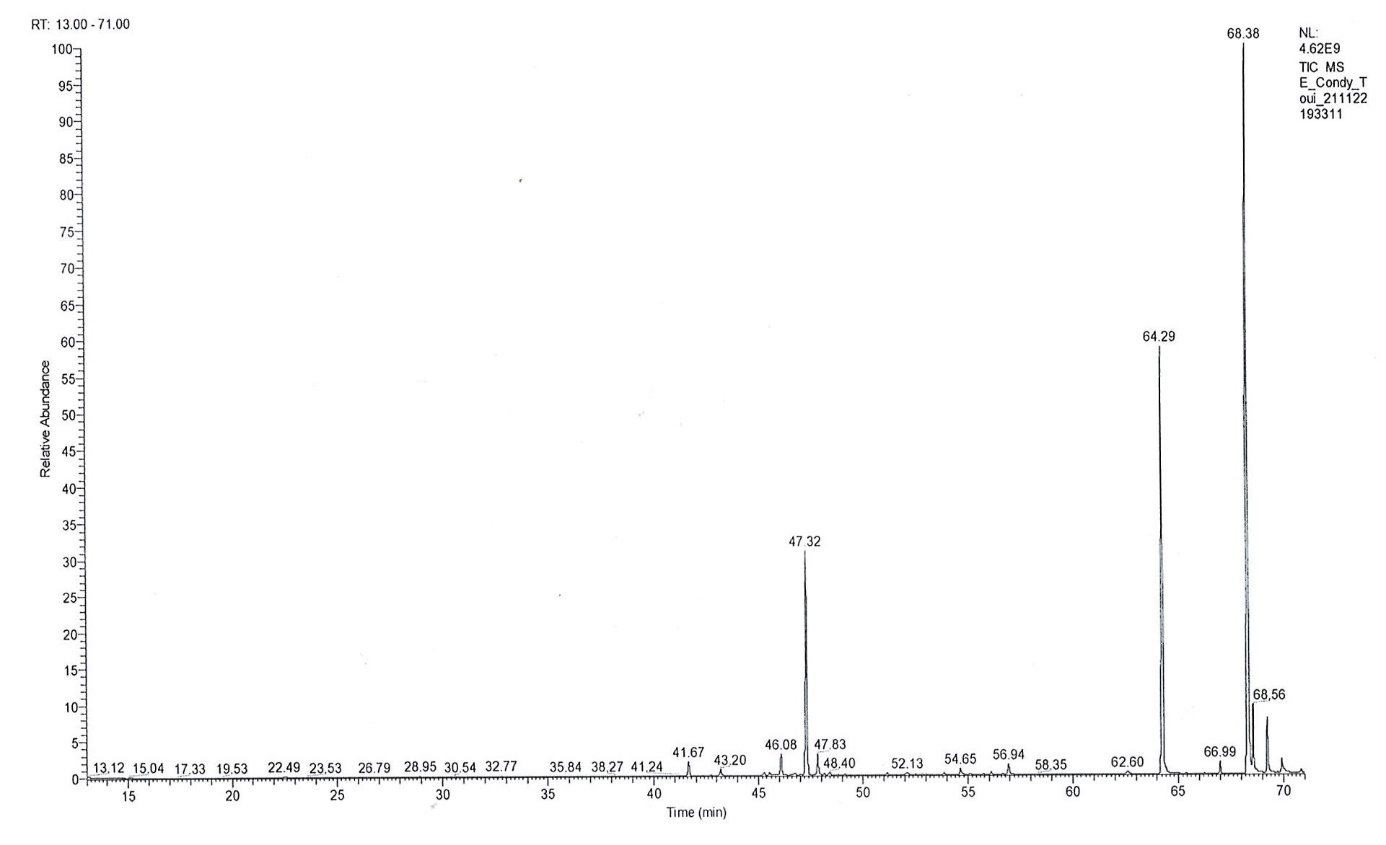


**S2 Fig** : Representative GC–MS chromatogram of Eu. Condylotricha flowers essential oil from Ouessè


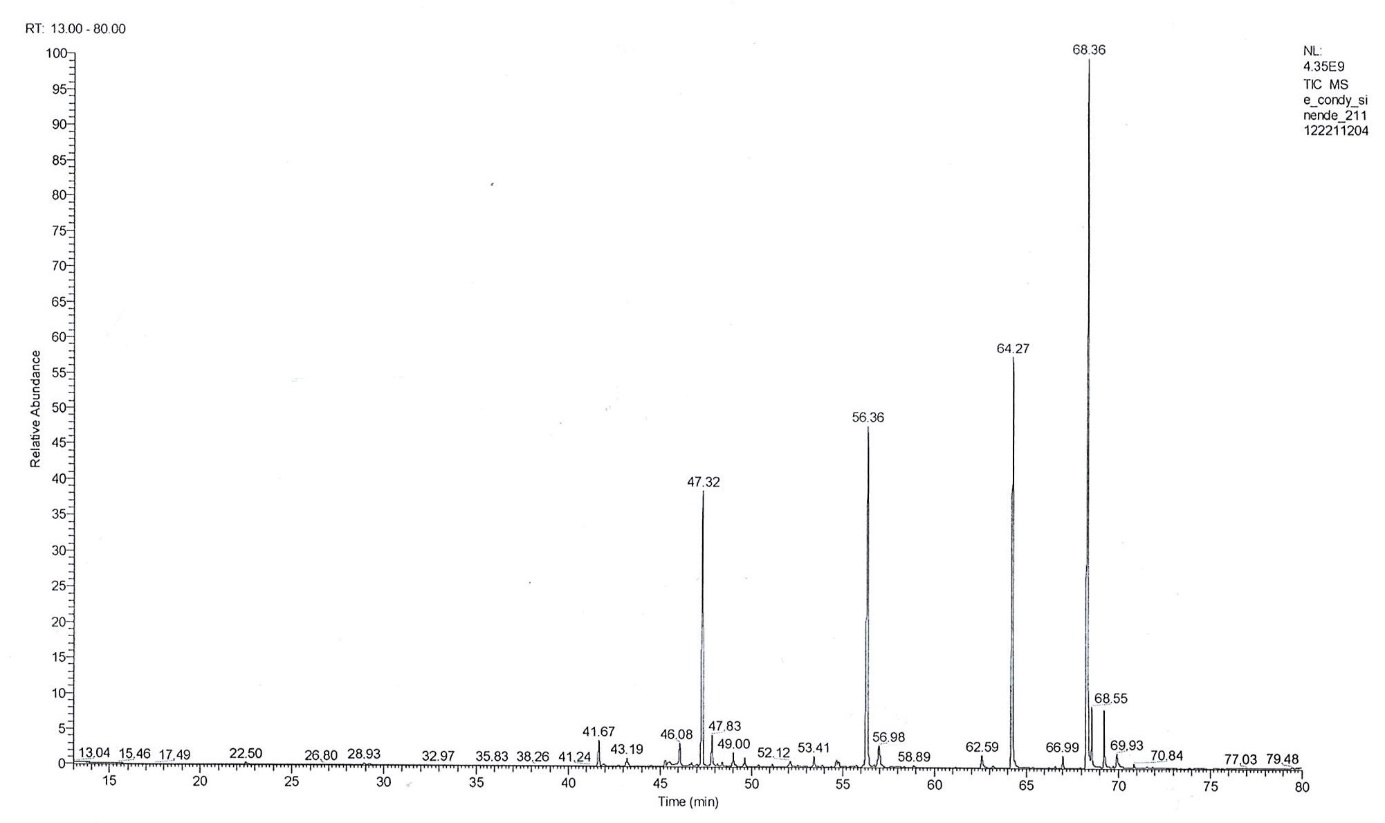


**S3 Fig**: Representative GC–MS chromatogram of Eu. Condylotricha flowers essential oil from Sinendé
