# Supplementary material for: Euclasta condylotricha flowers essential oils: A new source of juvenile hormones and its larvicidal activity against Anopheles gambiae s.s. (Diptera: Culicidae)
Source: PLoS One. 2023 Jan 23;18(1):e0278834. doi: 10.1371/journal.pone.0278834 (PMC9870135; doi:10.1371/journal.pone.0278834)
Supplement: Attachment — Submitted filename: Response to reviewers.pdf [file pone.0278834.s003.pdf]

Dear Editor,

Please find below the responses addressing each of the comments of the reviewers assigned to our submitted manuscript entitled: *Euclasta condylotricha* flowers essential oils: a new source of juvenile hormones and its larvicidal activity against *Anopheles gambiae s.s.* (Diptera: Culicidae).

Editor

1. Please ensure that your manuscript meets PLOS ONE's style requirements, including those for file naming. The PLOS ONE style templates can be found at https://journals.plos.org/plosone/s/file?id=wjVg/PLOSOne_formatting_sample_main_body.pdf and https://journals.plos.org/plosone/s/file?id=ba62/PLOSOne_formatting_sample_title_authors_affiliations.pdf

Reply: We reviewed again the journal formatting guidelines and ensured that the manuscript meets the style requirements.

2. Please describe any permissions and permits you had to collect plant samples.

Reply: Please, kindly note that at the University of Abomey-Calavi, there is not yet a policy to deliver permission or permit before plant collection. The general rule at the University is that after plant collection, the author deposits a plant sample at the National Herbarium and gets a voucher number.
Furthermore, the plant species used in this study was collected in the field in its natural habitat. The plant is an annual species, very wide distributed that colonizes agricultural lands and vacant fields. In that case, no permission or permit is delivered prior to the plant samples collection.

3. Thank you for stating the following financial disclosure: "RBB received financial support through Wellcome Trust intermediate fellowship in public health and tropical medicine grant (N° 109917/Z/15/Z) awarded to LSD. The funders had no role in study design, data collection and analysis, decision to publish, or preparation of the manuscript."

At this time, please address the following queries:

a) Please clarify the sources of funding (financial or material support) for your study. List the grants or organizations that supported your study, including funding received from your institution.

Reply: RBB received material support through the grant awarded to LSD. No grants or organizations supported the study. No funding was received from our institution.

b) State what role the funders took in the study. If the funders had no role in your study, please state: “The funders had no role in study design, data collection and analysis, decision to publish, or preparation of the manuscript.”

Reply: The funders had no role in study design, data collection and analysis, decision to publish, or preparation of the manuscript.

c) If any authors received a salary from any of your funders, please state which authors and which funders.

Reply: No author received a salary from the funder.

d) If you did not receive any funding for this study, please state: "The authors received no specific funding for this work."

Reply: The authors received no specific funding for this work.

Please include your amended statements within your cover letter; we will change the online submission form on your behalf.

Reply: Amended statements are provided in the cover letter.

4. PLOS requires an ORCID iD for the corresponding author in Editorial Manager on papers submitted after December 6th, 2016. Please ensure that you have an ORCID iD and that it is validated in Editorial Manager. To do this, go to 'Update my Information' (in the upper left-hand corner of the main menu), and click on the Fetch/Validate link next to the ORCID field. This will take you to the ORCID site and allow you to create a new iD or authenticate a pre-existing iD in Editorial Manager. Please see the following video for instructions on linking an ORCID iD to your Editorial Manager account: https://www.youtube.com/watch?v=_xcclfuvtxQ.

   Reply: ORCID detail of the corresponding author was provided and validated in the Editorial Manager.

5. Please review your reference list to ensure that it is complete and correct. If you have cited papers that have been retracted, please include the rationale for doing so in the manuscript text, or remove these references and replace them with relevant current references. Any changes to the reference list should be mentioned in the rebuttal letter that accompanies your revised manuscript. If you need to cite a retracted article, indicate the article's retracted status in the References list and also include a citation and full reference for the retraction notice.

Reply: Done.

Reviewer #1

The authors thank the reviewer for his comments on the manuscript.

Lines 76-77. "Considering the concerted efforts to develop plant products-based insecticides as an excellent alternative to synthetic insecticides,.." It is an interesting idea that has been proposed long time ago, the question is, how much (or how we evaluate) non-target species could be affected in their habitats by the use of such strategies?

Reply: This is a question that needs further investigations before implementing such strategy base on plant products. The final formulation of the plant-based products to be applied to the larval habitats need to be determined. The choice of an appropriate formulation will minimize the non-target impacts. To evaluate the effect of such strategy on non-target species, the final formulation to be delivered in the field should assess the toxicological effects of treating representative, sensitive indicator species, such as an aquatic invertebrates and vertebrates with the plant product.
Furthermore, since plant-based products are natural and eco-friendly materials, we assume that these products has no greater toxicological effects than that from the actual chemical larvicides.

Line 169. "…whole plants of Cyperus iria L.", should say "…whole plants of Cyperus iria L. and C. aromaticus Ridl."

Reply: *C. aromaticus* Ridl was added at the end of the sentence. Done.

Line 184. "…EOs…" instead "…essential oils"

Reply: *Done*

Lines 245-247. Clearly, results show that EOs have a larvicidal effect, but there is not a direct prove that such effect was by JH. It is only an association because our knowledge that JH III interfere with larvae development, but in EOs more components are present and no evaluations separately were performed. Conclusion (and/or discussion) should be clear on those limitations.

Reply: Please, notice that in Lines 245-247, we did not mean that the larvicidal effect of the EOs is due to the Juvenile Hormone. As conclusion, we were reporting the major phytochemical compound found in all EOs. In the discussion session, lines 225-241, we stated that the larvicidal effect could be due to the secondary metabolites present in the EOs (Juvenile hormone and sesquiterpenes). However, we added a statement in the conclusion. Please see line 249 to 252 as follow:

"*…Before the translation of these research findings into operational interventions, further investigations on mechanisms by which EOs mediate their bioinsecticidal activity may require evaluations of the major EOs' constituents separately…*"

Reviewer #2:

We are grateful to the reviewer for his comments on the manuscript.

Line 73: Proper spelling of the scientific name "Andropogon condylotrichus" remove the capital letter of the species.

Reply: Done.

Line 195: The value 35.54 does not appear in table 2; correct for the correct data.

Reply: Done.

Line 197-198: It should be: with LC50 of 38.10 ppm and 198 38.46 ppm....

Reply: Done.

Figure 2. The title should be "Percentage of dead An. gambiae larvae..." instead of "Number of dead An. gambiae larvae...". In the figures, the graphs and text are not clear, I suggest that they be replaced by figures of higher image quality.

Reply: The Title of Figure 2 was changed accordingly. The low quality of the Figure 2 may be due to the PDF generation system in the Editorial Manager. Otherwise, the figure 2 was submitted to PACE platform according to PLOS One recommendation for Figures. After figure correction using PACE (https://pacev2.apexcovantage.com/), the generated file was submitted with the manuscript. Therefore we assume that, if accepted for publication, the figure will be more clear on the online version of the paper. A new version of Figure 2 was submitted.
